# Distinct sperm nucleus behaviors between genotypic and temperature-dependent sex determination males are associated with replication and expression-related pathways in a gynogenetic fish

**DOI:** 10.1186/s12864-018-4823-6

**Published:** 2018-06-05

**Authors:** Yao-Jun Zhu, Xi-Yin Li, Jun Zhang, Zhi Li, Miao Ding, Xiao-Juan Zhang, Li Zhou, Jian-Fang Gui

**Affiliations:** 1 State Key Laboratory of Freshwater Ecology and Biotechnology, Institute of Hydrobiology, The Innovative Academy of Seed Design, Chinese Academy of Sciences, University of Chinese Academy of Sciences, Wuhan, 430072 China; 2 Institute of Hydrobiology, Chinese Academy of Sciences, Wuhan, 430072 China

**Keywords:** Genotypic sex determination, Temperature-dependent sex determination, Semen proteomics, Gynogenesis

## Abstract

**Background:**

Coexistence and transition of diverse sex determination strategies have been revealed in some ectothermic species, but the variation between males caused by different sex determination strategies and the underlying mechanism remain unclear. Here, we used the gynogenetic gibel carp (*Carassius gibelio*) with both genotypic sex determination (GSD) and temperature-dependent sex determination (TSD) strategies to illustrate this issue.

**Results:**

We found out that males of GSD and TSD in gibel carp had similar morphology, testicular histology, sperm structure and sperm vitality. However, when maternal individuals were mated with males of GSD, sperm nucleus swelling and fusing with the female pronucleus were observed in the fertilized eggs. On the contrary, when maternal individuals were mated with males of TSD, sperm nucleus remained in the condensed status throughout the whole process. Subsequently, semen proteomics analysis unveiled that DNA replication and gene expression-related pathways were inhibited in the sperm from males of TSD compared to males of GSD, and most differentially expressed proteins associated with DNA replication, transcription and translation were down-regulated. Moreover, via BrdU incorporation and immunofluorescence detection, male nucleus replication was revealed to be present in the fertilized eggs by the sperm from males of GSD, but absent in the fertilized eggs by the sperm from males of TSD.

**Conclusions:**

These findings indicate that DNA replication and gene expression-related pathways are associated with the distinct sperm nucleus development behaviors in fertilized eggs in response to the sperm from males of GSD and TSD. And this study is the first attempt to screen the differences between males determined via GSD and TSD in gynogenetic species, which might give a hint for understanding evolutionary adaption of diverse sex determination mechanisms in unisexual vertebrates.

**Electronic supplementary material:**

The online version of this article (10.1186/s12864-018-4823-6) contains supplementary material, which is available to authorized users.

## Background

Sex determination is amazingly plastic in vertebrates, and two kinds of contrasting strategies including genotypic sex determination (GSD) and environmental sex determination (ESD) have been extensively revealed [1–3]. The primary sex of individuals with GSD is determined at the moment of fertilization via genotypic elements with sex difference [4], and diverse systems of GSD have been identified such as male heterogametic XX/XY system, female heterogametic ZZ/ZW system and their numerous variants [1]. While individuals with ESD do not have genetic difference between sexes, and their sex is determined during development [4] via environmental factors including temperature [5], photoperiod [6], social factors [7], pH and dissolved oxygen (DO) [8]. These two seemingly distinct sex determination strategies are not mutually exclusive, and coexistence, interaction and transition of GSD and ESD have been observed in fishes [9, 10], amphibians [11] and reptiles [5]. Along with genomic anatomy and investigation of non-model organisms, diverse sex determination systems and evolutionary mechanisms of transition among sex determination systems have been unveiled gradually [1, 2, 5], but the variation within a sex between different sex determination strategies and the underlying mechanism remain unclear.

Gibel carp (*Carassius gibelio*) with a wide geographic distribution in Eurasian continent and neighboring islands [12–14], has two rounds of polyploidy origins including an ancient allopolyploidy and a recent autopolyploidy [13, 15]. Compared with other unisexual vertebrates [16], rare but significant male incidences were observed in many natural habitats [9, 17, 18] of gibel carp with unisexual gynogenetic ability [17, 19, 20]. Recently, both GSD and temperature-dependent sex determination (TSD) were found to coexist in gibel carp through analyses of natural populations and lab experimental progenies [9, 21]. Moreover, extra microchromosomes and high temperature were illustrated to play genotypic and environmental male determination role respectively [9, 21], and a possible association between sex determination mechanisms and reproduction modes were revealed [9]. Diverse sex determination mechanisms of GSD and TSD [9, 21] and dual reproduction modes including unisexual gynogenesis and sexual reproduction [17, 19, 20] make gibel carp an ideal system to investigate plastic sex determination strategies and their evolutionary consequences.

Here, we observed that there were no significant masculine characteristic differences between males of GSD and TSD in morphology and histology, and their sperms also had similar structure and vitality. However, distinct sperm nucleus development behaviors were revealed from the fertilized eggs by two kind sperms of GSD males and TSD males. Subsequently, we performed iTRAQ-based quantitative proteomics analyses of semen samples between GSD and TSD males. Compared to males of GSD, pathways assigned to DNA replication, transcription and translation were illustrated to be down-regulated in the sperm from males of TSD. Moreover, male nucleus replication was only revealed in the eggs fertilized by the sperm from males of GSD, while no male nucleus replication was detected in the eggs fertilized by the sperm from males of TSD, via BrdU incorporation and immunofluorescence detection. These results indicated that distinct sperm nucleus development behaviors between males of GSD and TSD were associated with DNA replication and gene expression-related pathways in gibel carp.

## Results

### Similar morphological masculine characteristics between males of GSD and TSD

**Fig. 1:**
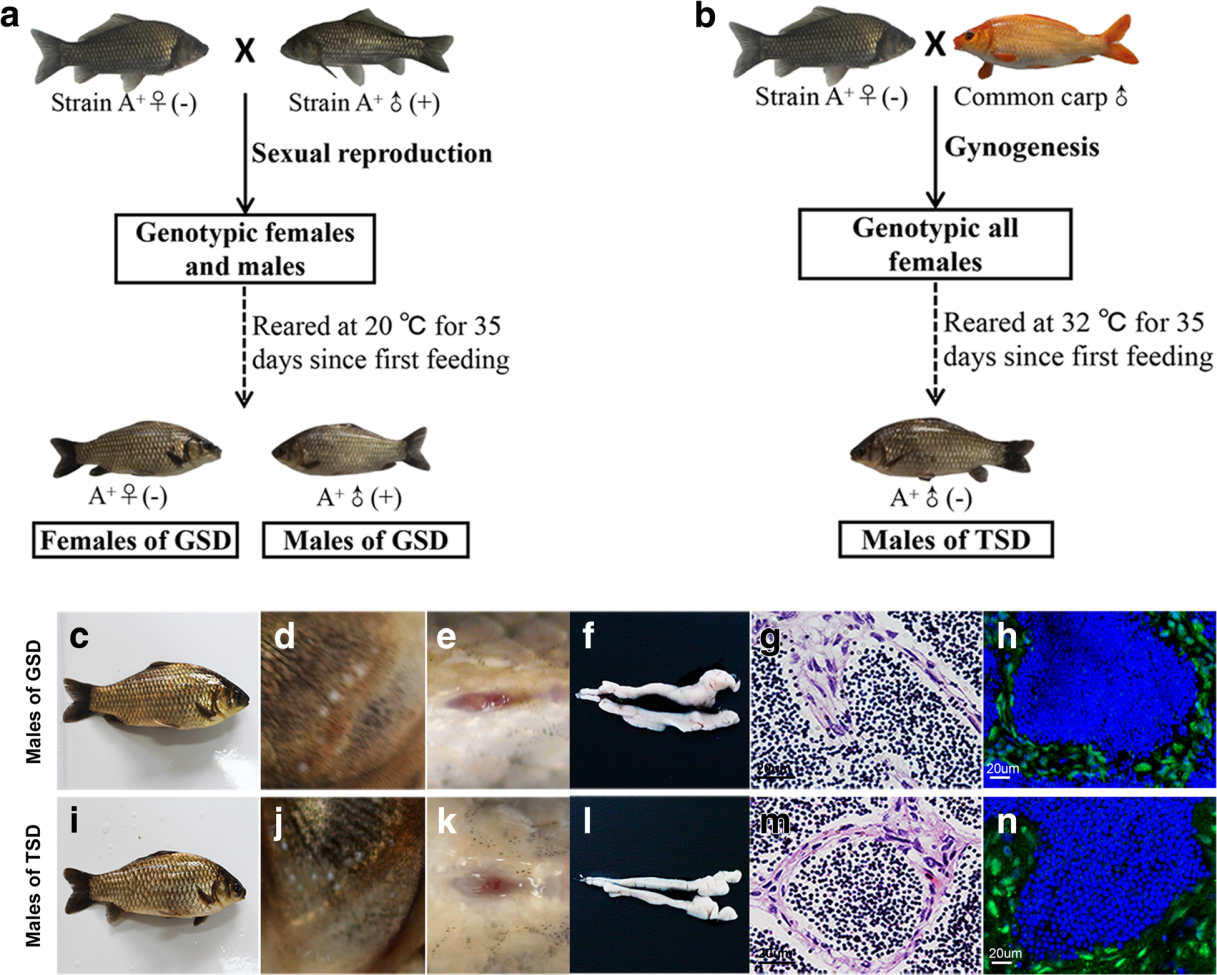
Cultivation, morphology and testicular histology of males determined via GSD and TSD. **a** Cultivation of males determined via GSD. **b** Cultivation of males determined via TSD. **c-n** Morphological masculine characteristics and testicular histology between males of GSD and TSD. **c**, **i** Body shape. **d**, **j** Gill cover. **e**, **k** Anus. **f**, **l** Testes. **g**, **m** Haematoxylin–eosin staining of testes. **h**, **n** Immunofluorescence staining of testes via *Cg*Vasa antibody. ♀: female; ♂: male; (+): with male-specific marker (MSM); (−): without MSM

Males of gibel carp are able to be determined via both genotypic extra microchromosomes and larval rearing temperature, respectively [9, 21]. So males of GSD with the genotypic male-specific marker (MSM) [9, 21] could be generated via sexual reproduction under normal rearing temperature at about 20 °C (Fig. 1a), while males of TSD without MSM [9] could be produced through high rearing temperature treatment (32 °C) of gynogenetic larvae (Fig. 1b). Through comparative investigation, we found out that both males of GSD and males of TSD showed normal male secondary sex characteristics during breeding season, such as slender body shape (Fig. 1c and i), pearl organs on gill cover (Fig. 1d and j) and prolate anus (Fig. 1e and k). Moreover, mature testis (Fig. 1f and l) with spermatogenic cysts (Fig. 1g and m) and numerous sperms (Fig. 1h and n) were also observed in both males of GSD and TSD.

### Similar sperm morphology, structure, vitality, and hatchability

**Fig. 2:**
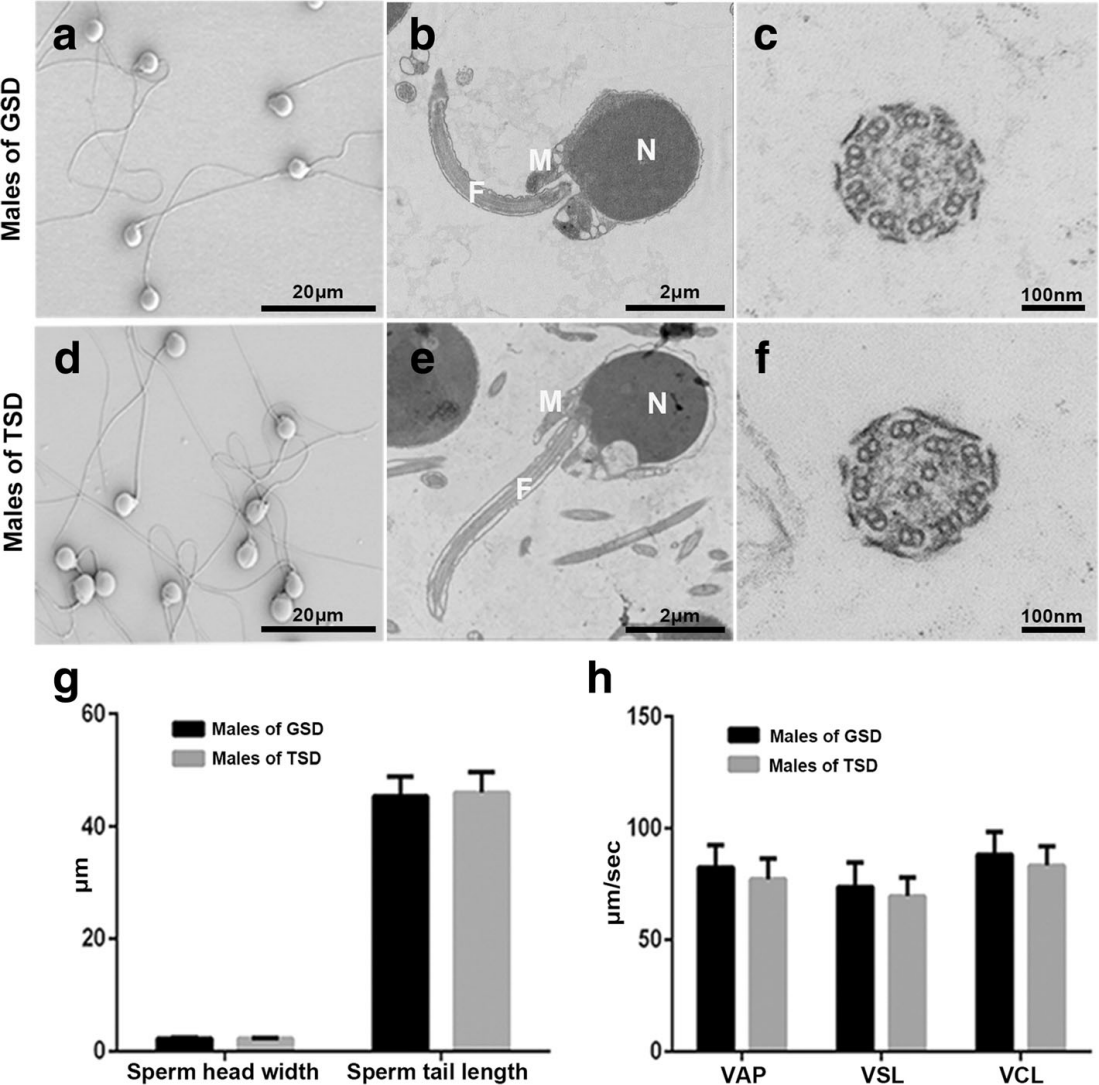
Comparisons of sperm structure, size and vitality between males of GSD and TSD. SEM (**a**, **d**) and TEM (**b**, **c**, **e**, **f**) analysis of sperm from males of GSD and TSD. N: nucleus; M: mid-piece; F: flagellum. **g** Statistical data of sperm head width and sperm tail length. **h** Statistical data of sperm vitality. VAP: average path velocity, VSL: straight-line velocity, VCL: curvilinear velocity. Vertical bars: standard deviation

To reveal similarities and differences between the sperm from males of GSD and TSD, we firstly examined the sperm morphology under scanning electron microscope (SEM) and both types of sperm had normal morphology that a flagellum came from the basal body (Fig. 2a and d). The sperm structure with nucleus (N), mid-piece (M) and flagellum (F) were observed (Fig. 2b and e), and the flagellum composed of 9 doublet microtubules and 2 central microtubules were also detected in both males of GSD and TSD (Fig. 2c and f) under transmission electron microscopy (TEM). Moreover, sperm head width and sperm tail length were 2.34 ± 0.02 μm and 45.44 ± 0.55 μm in males of GSD as well as 2.29 ± 0.02 μm and 46.03 ± 0.61 μm in males of TSD, respectively (T-test: *P* = 0.10 and *P* = 0.48, respectively) (Fig. 2g). Subsequently, to unveil sperm vitality, we analyzed average path velocity (VAP) (82.52 ± 2.39 μm/sec), straight-line velocity (VSL) (73.84 ± 2.66 μm/sec) and curvilinear velocity (VCL) (88.26 ± 2.50 μm/sec) of sperm from males of GSD, which were not significantly different from the VAP (77.23 ± 2.07 μm/sec), VSL (69.56 ± 1.91 μm/sec) and VCL (83.25 ± 1.98 μm/sec) of sperm from males of TSD (T-test: *P* = 0.10, *P* = 0.19 and *P* = 0.12, respectively) (Fig. 2h). Besides, there were also no significant differences on hatchability between eggs fertilized by the sperm from males of GSD (59% ± 23%) and TSD (68% ± 17%) (T-test: *P* = 0.76). These results indicate that there are no significant differences in sperm morphology, structure, vitality, and hatchability between males of GSD and TSD.

### Different male incidence

**Fig. 3:**
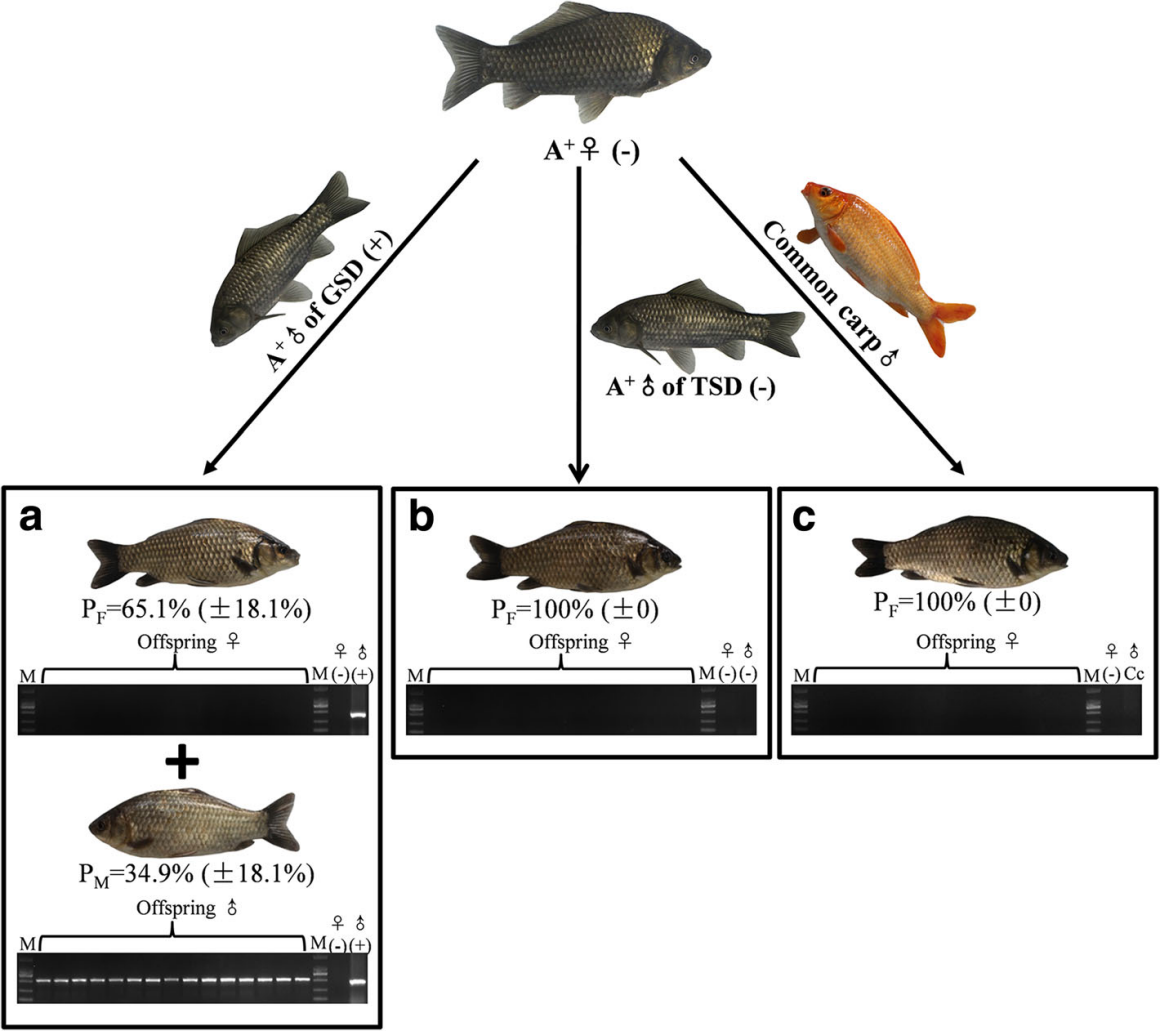
Male incidence in the offspring. The maternal individuals from strain A^+^ were mated with males of GSD (**a**), males of TSD (**b**) and another species common carp (**c**). PCR detection analysis of MSM in the randomly-picked offspring and the parental individuals was shown at the bottom. ♀: female; ♂: male; (+): with MSM; (−): without MSM; P_M_: male proportion; P_F_: female proportion; Cc: Common carp; M: DL2000 marker. Detail data of replicates are given in additional file 1: Table S1

Although there were many similarities between males of GSD and TSD, male incidences in the offspring were revealed to be distinct between these two types of males. When the maternal fish from strain A^+^ were mated with males of GSD from strain A^+^, 34.9% (±18.1%) males were detected in the offspring, and the MSM [21] was present in all sampled males and the paternal individuals, whereas it was absent in all randomly picked females and the maternal individuals (Fig. 3a and Additional file 1: Table S1). However, when maternal fish were mated with males of TSD from strain A^+^, no male offspring was generated and the MSM was absent in all randomly picked females and the parental individuals (Fig. 3b and Additional file 1: Table S1), which were identical to the gynogenetic families (Fig. 3c and Additional file 1: Table S1). The similar results were also revealed previously in the wild population sampled from Poyang Lake in China [9].

### Distinct sperm nucleus events and development behaviors

**Fig. 4:**
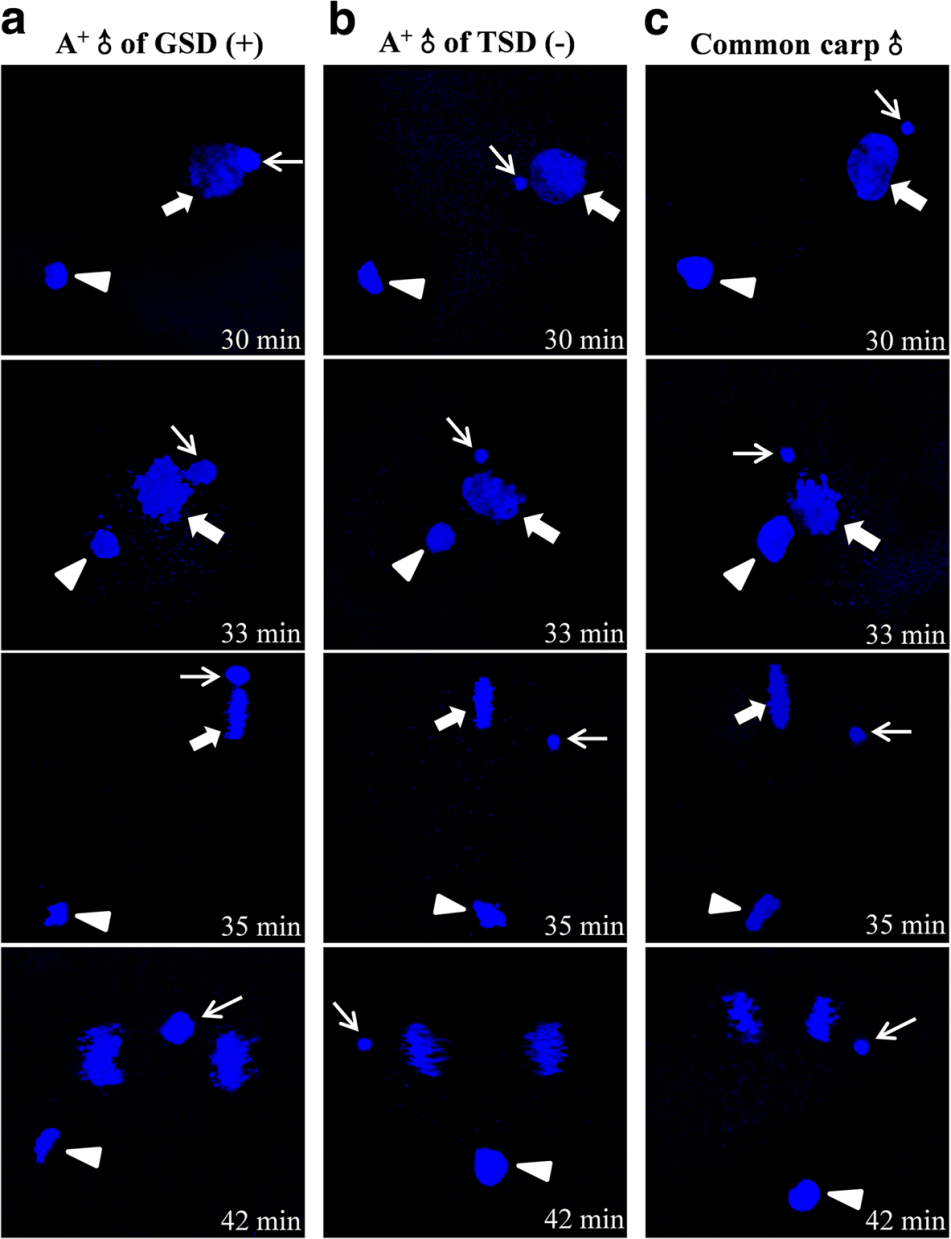
DAPI-stained differential male nucleus behaviors in fertilized eggs. The fertilized eggs of strain A^+^ inseminated by the sperms from males of GSD (**a**), males of TSD (**b**) and another species common carp (**c**). Thin arrows indicate sperm nucleus or male pronucleus. Thick arrows indicate female pronucleus. Arrowheads indicate second polar-body. Time showed on the right corner is the corresponding time after fertilization

Moreover, differential male nucleus events and development behaviors were also revealed from the fertilized eggs in response to the sperm from males of GSD and TSD. When the eggs of maternal fish were fertilized by the sperm from males of GSD, the fertilized egg encountered similar sexual reproduction events and behaviors, including sperm nucleus swelling, male pronucleus fusing with female pronucleus before 33 min, even though male pronucleus did not completely integrate into the first mitosis after 35 min (Fig. 4a). However, when the eggs of maternal fish were fertilized by the sperm from males of TSD, the entered sperm nucleus was always preserved in the condensed status throughout the whole first mitosis process (Fig. 4b), which was identical to a typical process of gynogenesis stimulated by heterologous sperm from common carp (Fig. 4c). The data indicate that there exist distinct sperm nucleus events and development behaviors in the fertilized eggs between GSD and TSD males, in which the sperm of GSD males undergoes sperm nucleus swelling and pronucleus fusing similar to sexual reproduction, whereas the sperm of TSD males only triggers a typical unisexual gynogenesis.

### Comparative semen proteomics and differentially expressed protein identification

**Fig. 5:**
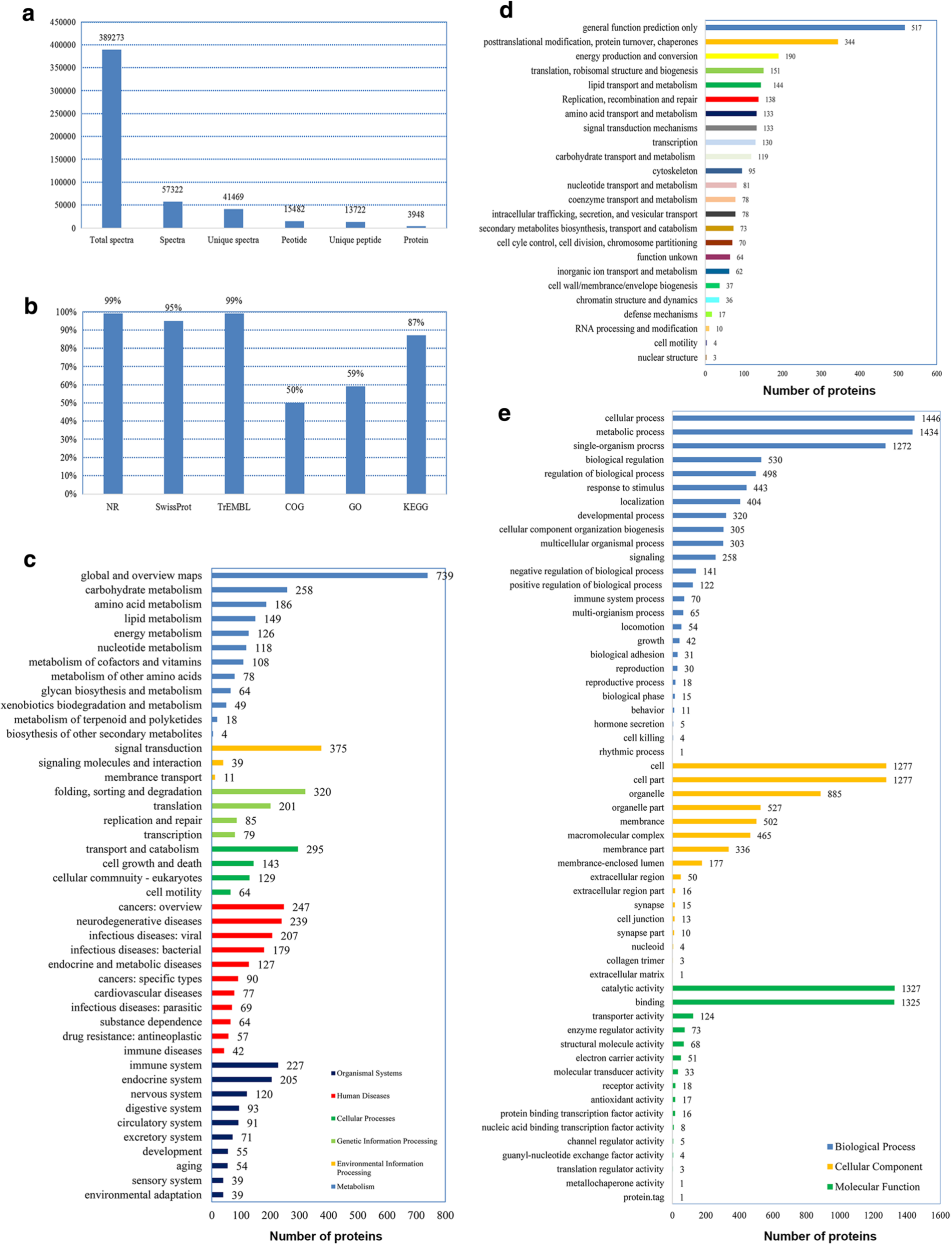
Summary information of semen proteomics. a Statistics of semen proteomic sequencing. **b** Overview of unigene/contig annotation in 6 databases including NR, Swiss-Prot, TrEMBL, COG, GO and KEGG. **c** Histogram of KEGG classification. **d** Histogram of COG classification. **e** Histogram of GO classification

To reveal the underlying molecular mechanism of differential sperm nucleus development behaviors in the fertilized eggs, iTRAQ-based quantitative semen proteomics were performed on three semen samples from three males of GSD and three semen samples from three males of TSD respectively. A total of 13,722 unique peptides and 3948 proteins were identified from 389,273 spectra (Fig. 5a and Additional file 2: Table S2). Subsequently, 99.2% (3916 proteins) of these proteins were annotated via any of the 6 databases including non-redundant protein (NR), Swiss-Prot, Translated EMBL Nucleotide Sequence Data Library (TrEMBL), Clusters of Orthologous Groups of proteins (COG), Gene Ontology (GO) and Kyoto Encyclopedia of Genes and Genomes (KEGG) (Fig. 5b and Additional file 2: Table S2). In the KEGG classification, 3436 proteins were mapped to 44 terms belonged to 6 main categories (Fig. 5c). The pathway with most annotated proteins was “global and overview maps” (739 proteins), followed by “signal transduction” (375 proteins), “folding, sorting and degradation” (320 proteins), “transport and catabolism” (295 proteins), and so on. Besides, 1972 proteins were classified into 24 COG terms, such as “general function prediction only”, “posttranslational modification, protein turnover, chaperones”, “energy production and conversion”, “replication, recombination and repair” and so on (Fig. 5d). And 2345 proteins were classified into 57 GO terms, and “cellular process”, “cell” and “catalytic activity” were dominant in the category of “biological process”, “cellular component” and “molecular function”, respectively (Fig. 5e). Compared to semen proteomics from males of GSD, 753 differentially expressed proteins(DEPs)were identified (fold change > 1.2, *P* < 0.05) in semen proteomics from males of TSD, including 310 up-regulated DEPs (Additional file 3: Table S3) and 443 down-regulated DEPs (Additional file 4: Table S4).

### Revelation of differentially regulated pathway

**Fig. 6:**
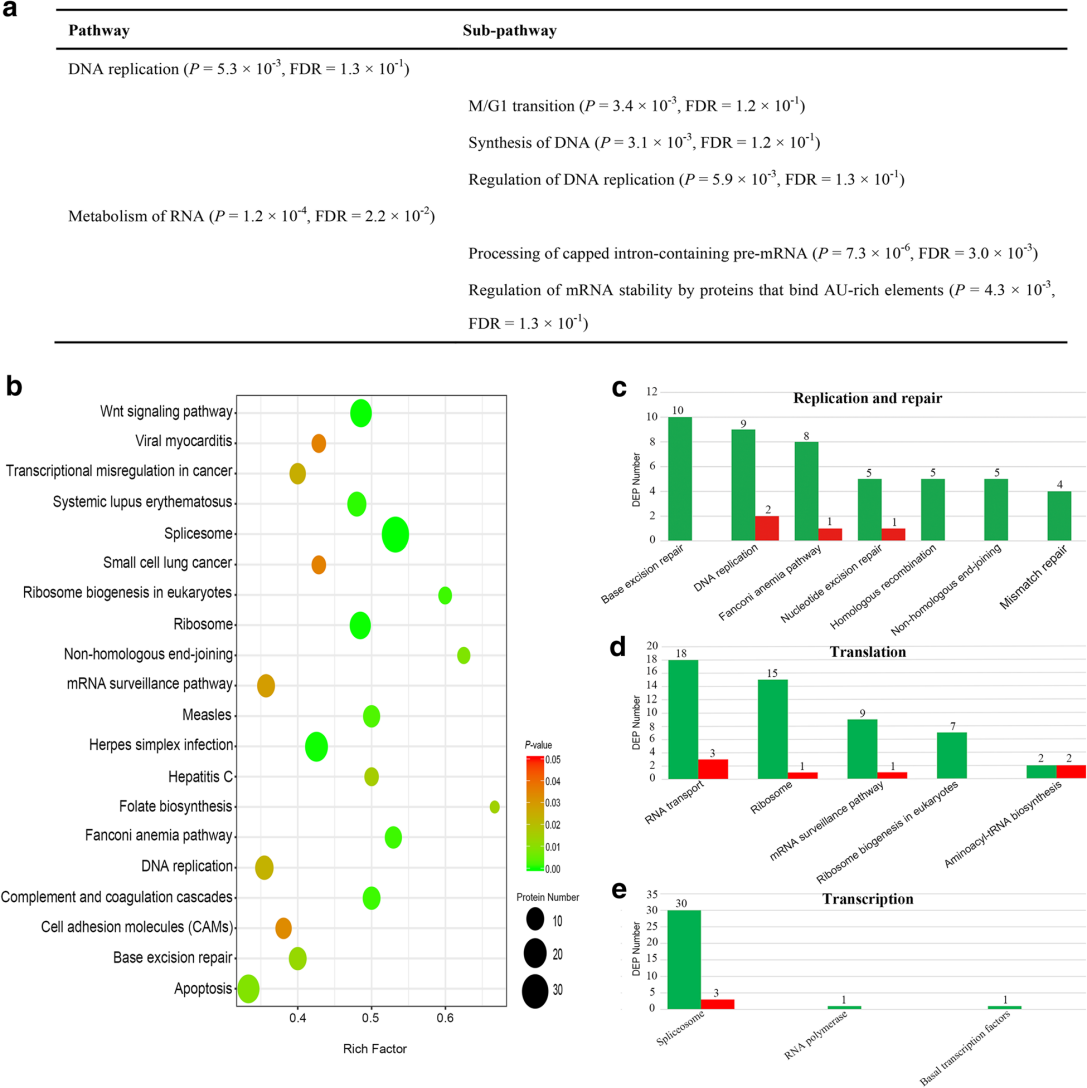
Analysis of differentially regulated pathway. **a** Down-regulated biological pathways in the sperm from males of TSD via *Reactome* database overrepresentation analysis. **b** Top 20 enriched KEGG pathways of DEPs in males of TSD compared with males of GSD. The x-axis indicates the rich factor of each pathway and y-axis shows pathway. Detail data of the 20 pathways are given in the additional file 5: Table S5. **c** Histogram of DEPs assigned to the 7 pathways belonged to the process of “Replication and repair”. **d** Histogram of DEPs assigned to the 5 pathways belonged to the process of “Translation”. **e** Histogram of DEPs assigned to the 3 pathways belonged to the process of “Transcription”. Red and green columns indicate up-regulated and down-regulated DEPs respectively. Detail data of DEPs are given in the Additional file 6: Table S6, Additional file 7: Table S7 and Additional file 8: Table S8 respectively, related to Fig. 6c, d and e respectively

To identify the biological pathways that might be differentially expressed in the semen between males of TSD and GSD, all the DEPs were used to performed overrepresentation analysis via *Reactome* database [22–25], only “DNA replication” and “Metabolism of RNA” were revealed to be down-regulated pathways based on *P* < 0.01 and the False Discovery Rate (FDR) < 0.25 [22, 26]. And, all 3 sub-pathways assigned to the pathway of “DNA replication”, such as “M/G1 transition”, “Synthesis of DNA” and “Regulation of DNA replication”, were detected as down-regulated sub-pathways (FDR = 0.12, 0.12 and 0.13, respectively). And, two sub-pathways in the pathway of “Metabolism of RNA”, such as “Processing of capped intron-containing pre-mRNA” and “Regulation of mRNA stability by proteins that bind AU-rich elements”, were illustrated to be down-regulated (FDR = 0.003 and 0.13, respectively) (Fig. 6a).

Subsequently, pathway enrichment analysis revealed that these 753 DEPs were mapped to 275 pathways through KEGG classification, and the top 20 enriched pathways were showed in Fig. 6b. Among the top 20 enriched pathways, the process of “Replication and repair” contained the most pathways, such as “Fanconi anemia pathway”, “Non-homologous end-joining”, “Base excision repair” and “DNA replication” (Fig. 6b and Additional file 5: Table S5), and the process of “Translation” had three enriched pathways including “Ribosome biogenesis in eukaryotes”, “Ribosome” and “mRNA surveillance pathway”, while the pathway of “Spliceosome” belonged to the process of “Transcription” had the most DEPs (Fig. 6b and Additional file 5: Table S5).

In the process of “Replication and repair”, a total of 27 DEPs were identified to include 24 (88.9%) down-regulated DEPs and 3 (11.1%) up-regulated DEPs. There were 10, 9, 8, 5, 5, 5 and 4 down-regulated DEPs in the pathway of “Base excision repair”, “DNA replication”, “Fanconi anemia pathway”, “Nucleotide excision repair”, “Homologous recombination”, “Non-homologous end-joining” and “Mismatch repair” respectively, while only 2, 1 and 1 up-regulated DEPs were identified in the pathway of “DNA replication”, “Fanconi anemia pathway” and “Nucleotide excision repair” respectively (Fig. 6c and Additional file 6: Table S6). The process of “Translation” contains 41 (87.2%) down-regulated DEPs and 6 (12.8%) up-regulated DEPs. There were 18, 15, 9, 7 and 2 down-regulated DEPs in the pathway of “RNA transport”, “Ribosome”, “mRNA surveillance pathway”, “Ribosome biogenesis in eukaryotes” and “Aminoacyl-tRNA biosynthesis” respectively, while only 3, 1, 1 and 2 up-regulated DEPs were detected in the pathway of “RNA transport”, “Ribosome”, “mRNA surveillance pathway” and “Aminoacyl-tRNA biosynthesis” respectively (Fig. 6d and Additional file 7: Table S7). And 35 DEPs were identified in the process of “Transcription” to include 32 (91.4%) down-regulated DEPs and 3 (8.6%) up-regulated DEPs. There were 30, 1 and 1 down-regulated DEPs in the pathway of “Spliceosome”, “RNA polymerase” and “Basal transcription factors” respectively, while only 3 up-regulated DEPs were revealed in the pathway of “Spliceosome” (Fig. 6e and Additional file 8: Table S8). Thus, most DEPs associated with “Replication and repair”, “Translation” and “Transcription” were down-regulated in the semen from males of TSD compared with males of GSD, which was consistent with the results of overrepresentation analysis via *Reactome* database (Fig. 6a).

Although many proteins have been identified from the seminal plasma in rainbow trout (*Oncorhynchus mykiss*) [27] and common carp (*Cyprinus carpio*) [28], the proteins involved in the DNA replication and gene expression were detected only in sperm proteins instead of the seminal plasma proteins [29]. And all down-regulated DEPs assigned to “Replication and repair”, “Translation” and “Transcription” had intracellular distributions (Additional file 9: Table S9), which indicated that these DEPs in semen were identified from sperm proteins. These findings suggest that most of the DNA replication and gene expression-related proteins should be disturbed in the sperm from males of TSD compared to males of GSD. Thereby, we propose a hypothesis that DNA replication, translation and transcription-related pathways might be associated with the distinct sperm nucleus behaviors between males of GSD and TSD.

### Confirmation of sperm nucleus replication difference

**Fig. 7:**
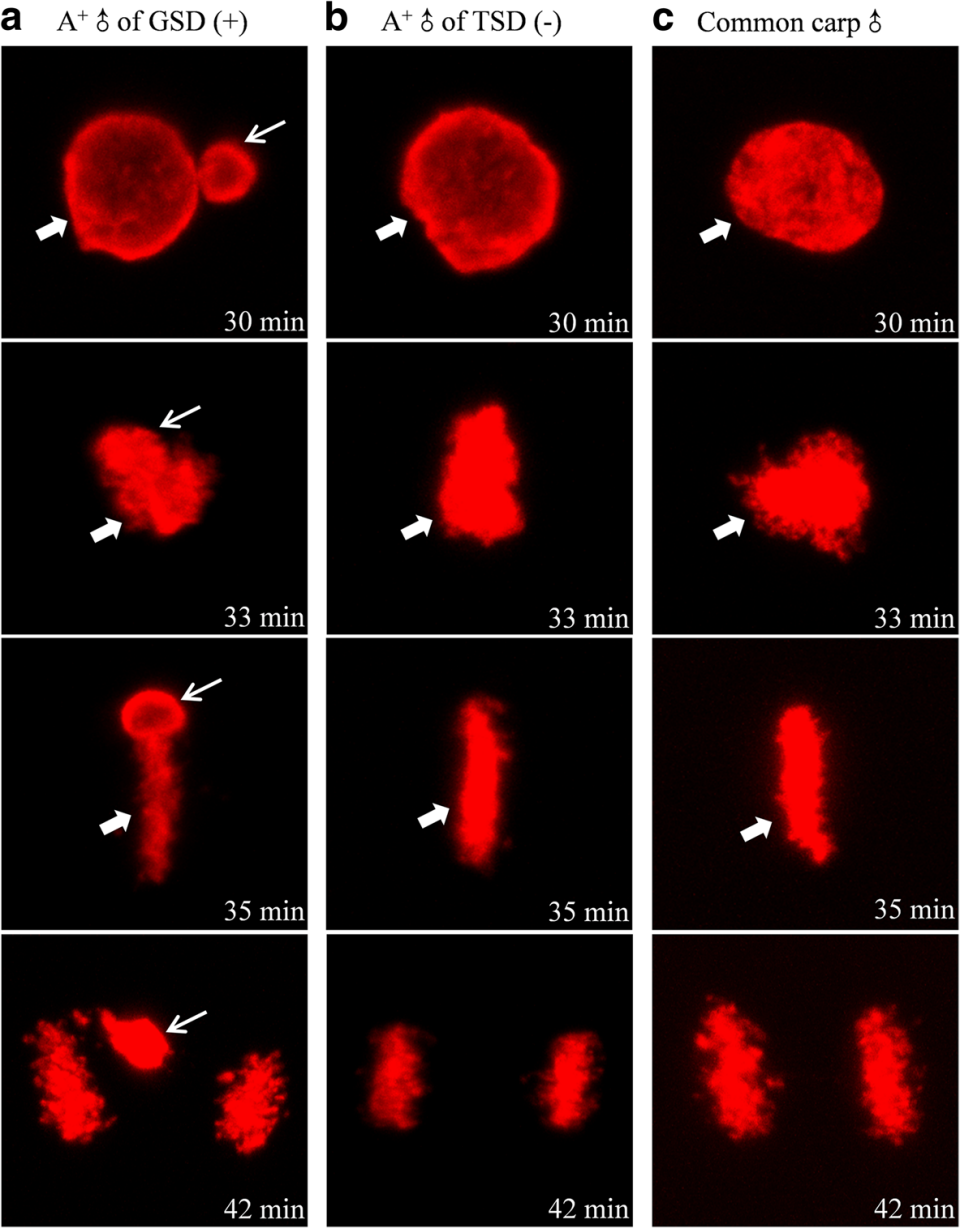
BrdU incorporation-marked distinct DNA replication of male pronucleus in fertilized eggs. The fertilized eggs of strain A^+^ inseminated by the sperms from males of GSD (**a**), males of TSD (**b**) and another species common carp (**c**). Thin arrows indicate the replicated male pronucleus and thick arrows indicate the replicated female pronucleus. Time showed on the right corner is the corresponding time after fertilization

To confirm the association between DNA replication and sperm nucleus development behaviors in the fertilized eggs, we used BrdU incorporation and immunofluorescence detection to trace DNA replication status as described previously [19]. When the eggs of maternal fish were fertilized by the sperm from males of GSD, both female and male nucleus experienced DNA replication as they migrated and contacted with each other. And the replicating pronuclei combined and formed zygote nucleus from 30 to 33 min, even though the replicated male chromatin bubble was divorced from the maternal chromosomes at 35 min and failed to integrate into the first zygotic mitosis at 42 min (Fig. 7a). However, when the eggs of the same maternal fish were fertilized by the sperm from males of TSD, no DNA replication signal of male nucleus was observed during the whole first mitosis, and only female pronucleus underwent genome replication and completed the first cleavage (Fig. 7b), which was identical to the typical gynogenetic process (Fig. 7c). And the absence of male nucleus replication in the fertilized eggs by the males of TSD might be associated with down-regulation of DNA replication proteins in the semen from males of TSD compared to males of GSD.

## Discussion

In this study, gibel carp males of genotypic and temperature-dependent sex determination were produced via sexual reproduction under normal rearing temperature and through high rearing temperature treatment of gynogenetic larvae, respectively. And males of GSD and TSD had similar morphology, testis histology, and sperm structure and sperm vitality. However, presence and absence of male nucleus swelling were observed in the fertilized eggs in response to the sperm from males of GSD and TSD, respectively. Subsequently, semen proteomics analysis revealed that DNA replication and gene expression-related pathways were down-regulated in the semen from males of TSD, compared to males of GSD. And the sperm from males of TSD was illustrated to have lower expression of most DEPs associated with DNA replication, transcription and translation than the sperm from males of GSD. Besides, male nucleus replication was only observed in the fertilized eggs by the sperm from males of GSD, which was not detected in the eggs fertilized by the sperm from males of TSD.

When the sperm from males of GSD fertilized the eggs of maternal gibel carp, sperm nucleus swelling was correlated with sperm nucleus replication (Fig. 4a and 7a). Meanwhile, when the sperm from males of TSD fertilized the eggs of maternal gibel carp, sperm nucleus without swelling was associated with absence of sperm nucleus replication (Fig. 4b and 7b). These results indicated that male nucleus swelling is closely related with the DNA replication of male nucleus. On the other hand, most proteins of the pathways assigned to DNA replication were revealed to be down-regulated in the sperm from males of TSD, compared to the males of GSD (Fig. 6). In *Caenorhabditis elegans*, DNA replication initiation was also reported to trigger rapid decondensation of chromatids during the first embryonic mitosis after fertilization [30]. Therefore, we deduce that down-regulation of DNA replication-related proteins in the sperm from males of TSD might be associated with the absence of male nucleus replication and swelling in the fertilized eggs in response to the sperm from males of TSD.

**Fig. 8:**
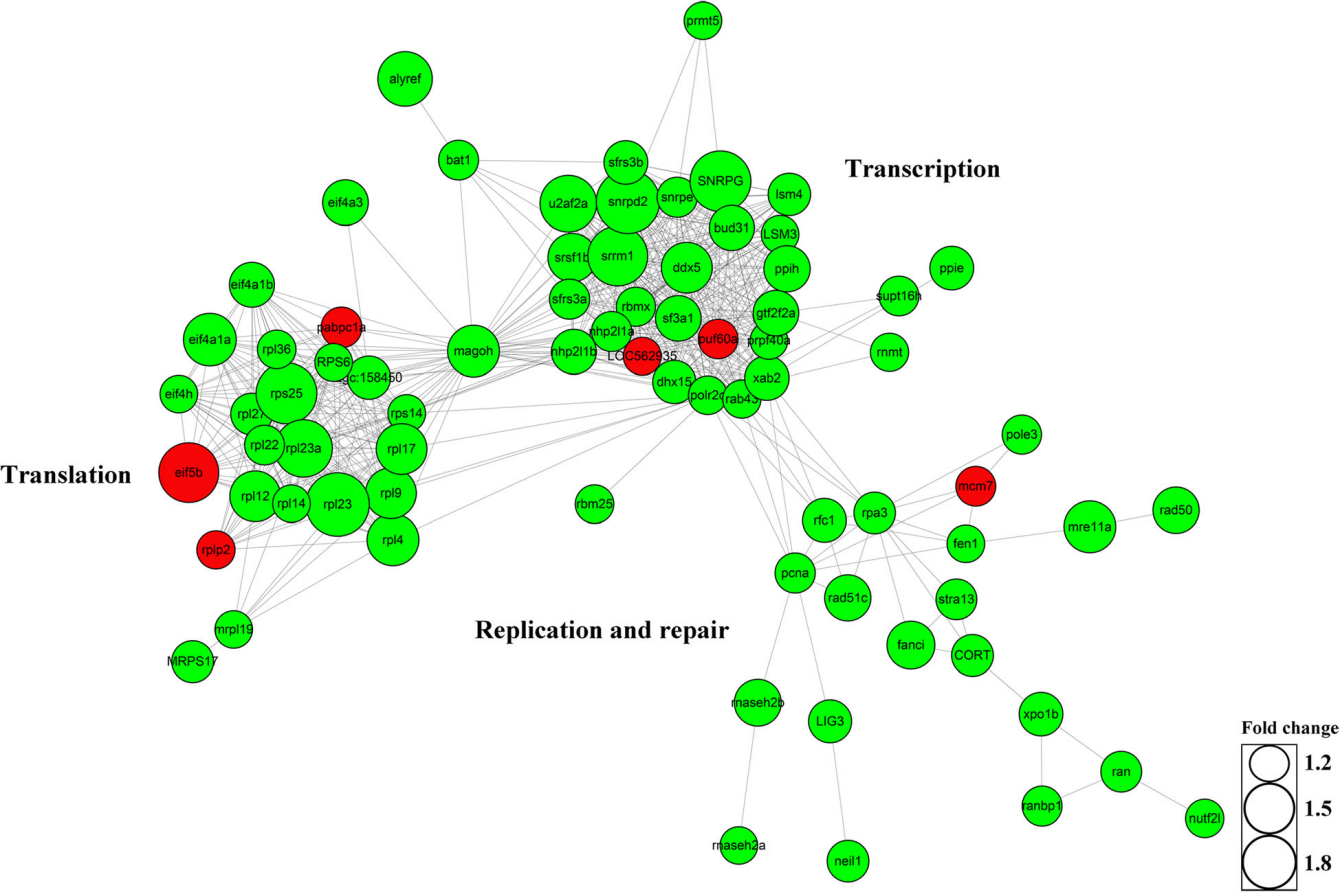
Network analysis for DEPs assigned to “Replication and repair”, “Translation” and “Transcription”. The fold changes of DEPs are presented with different size. Green color indicates down-regulated DEPs, while red color indicates up-regulated DEPs

In overrepresentation analysis via *Reactome* database, the pathway of “Metabolism of RNA” was also illustrated to be down-regulated except the pathway of “DNA replication” (Fig. 6a). And in the top 20 enriched KEGG pathways, the pathways belonged to “Translation” and “Transcription” were also down-regulated (Fig. 6b, d and e), which indicated that not only DNA replication-related pathway proteins were down-regulated, but also gene expression-related pathway proteins were inhibited in the sperm form males of TSD compared to males of GSD. In addition, most DEPs that are assigned to “Replication and repair”, “Transcription” and “Translation” are connected in the interaction network analysis (Fig. 8). Thus, down-regulation of gene expression-related proteins might be also associated with absence of male nucleus replication and swelling in the fertilized eggs by the sperm from males of TSD, though DNA replication was deduced to be critical to distinct male nucleus behaviors between males of TSD and GSD as discussed above.

Although sperm nucleus without replication and swelling were both observed in the fertilized eggs in response to the sperm from gibel carp males of TSD (Fig. 4b and 7b) and the heterologous sperm from common carp (Fig. 4c and 7c), the potential mechanisms might be different from each other. In many bisexual species, sperm nucleus swelling and replication were not species-specific, even between species with far relationship [31–34]. Previous investigations revealed that common carp sperm swelled and formed pronuclei obviously in the common carp egg extracts [35], and hybrids were easily produced between common carp males and other bisexual fish species [33, 34]. However, common carp sperm were not able to swell and form pronuclei in the gibel carp extracts [35], and a typical process of gynogenesis stimulated by the heterologous sperm was observed when maternal gibel carp were mated with common carp [19]. Thus, the condensation status of common carp sperm in the eggs of gibel carp might be caused by the inhibition from gibel carp eggs instead of common carp sperm per se.

Gynogenetic gibel carp has two rounds of polyploidy origins. The first polyploidy event might result in ancestral tetraploid *Carassius auratus* [15, 36] and the late round of multiple independent polyploidy events from sympatric tetraploid *C. auratus* might lead to the extant hexaploid gibel carp [13–15, 36]. The newly formed gibel carp with unisexual gynogynetic ability, might enter evolutionary trajectory of diploidization, as diploidization process after polyploidy was suggested to be the driving force of recurrent polyploidy [13, 37, 38]. Perhaps, it is the two rounds of polyploidy and diploidization as major evolutionary force that leads to different sex determination mechanisms of GSD and TSD, and acquires facultative reproduction strategies of unisexual and bisexual reproduction modes in the extant hexaploid gibel carp.

TSD is the most common system in ESD and occurs in very many reptiles [5, 11], some fishes [39, 40] and amphibians [11, 41]. In gibel carp, males of TSD trigger typical unisexual gynonenesis (Figs. 3, 4 and 7) which is able to achieve high fecundity [42], but TSD is commonly considered to be particularly vulnerable to climate change [43, 44]. Compared with TSD, males of GSD in gibel carp, which are able to stimulate reproduction mode similar to bisexual reproduction (Figs. 3, 4 and 7), should have more genetic contribution to the offspring than males of TSD (Fig. 3). And the proportions of GSD is much higher than that of TSD in sympatric population across mainland China [9]. Although some unisexual vertebrates have widespread ecological distribution and long existence scale, unisexual reproduction is suggested to be invariable failure as a long-term evolutionary strategy [45], that might be why most species reproduce by obligate bisexual reproduction [16, 42]. Thus, the hexaploid gibel carp might be also under the reproduction mode transition from unisexual gynogenesis to bisexual reproduction for a long evolutionary term [13, 19, 21], and males of GSD with the ability to stimulate reproduction mode similar to bisexual reproduction in the unisexual species might have more selective benefits than males of TSD with the ability to trigger unisexual gynogenesis.

## Conclusion

We have screened variations between males of GSD and TSD in gynogenetic gibel carp, and revealed that DNA replication and gene expression-related pathways are associated with the distinct male nucleus development behaviors in fertilized eggs in response to the sperm from males of GSD and TSD, which might help us with understanding evolutionary adaption of diverse sex determination mechanisms in unisexual vertebrates.

## Methods

### Experimental animal source

All experimental fish including gibel carp (*C. gibelio*) and common carp (*C. carpio*) were collected from Wuhan Guanqiao Experimental Station, Institute of Hydrobiology, Chinese Academy of Sciences. Strain A^+^ [46] of gibel carp and red common carp were used for the analyses in this study. The phenotypic sex of these fish were distinguished based on whether they ovulated and released eggs or produced sperm in the breeding season. Males of GSD were distinguished from males of TSD via the male-specific marker (MSM) identified previously [21].

### Artificial propagation and fish culture

In the breeding season of gibel carp, the selected maternal fish were induced into spawning by intraperitoneal injection as described previously [47]. About 8–10 h after injection, the maternal fish started to ovulate, and the ovulated eggs were inseminated with sperms from gibel carp or red common carp. The embryos were incubated in culture dishes at 23 °C (±1 °C) during the periods of embryogenesis and larval hatching, and then the hatched larvae were reared at 20 °C (±1 °C) in water boxes equipped with an inflator pump. The larvae were fed with fairy shrimp for 35 d since first feeding and then maintained in outdoor tanks (5 m × 4 m × 1.5 m) with normal feed.

To produce males of TSD, the embryos of gynogenetic family were firstly incubated in culture dishes at 23 °C (±1 °C) until first feeding, and then the larval rearing temperature were gradually changed to 32 °C (±1 °C) as previously described [9]. The larvae were reared in water boxes equipped with inflator pump during the larval rearing period for 35 d. At last the fry were also maintained in outdoor tanks with normal feed.

### Haematoxylin–eosin and immunofluorescence staining

The testes were dissected from males of GSD and males of TSD, and fixed in 4% paraformaldehyde in PBS overnight at 4 °C. Then samples were dehydrated and embedded in paraffin, and were cut into 5 μm sections. HE staining was performed as previously described [48] and immunofluorescence staining using *Cg*Vasa antibody was performed as described [49].

### Scanning electron microscope and transmission electron microscopy

The semen was fixed in PBS buffer (pH = 7.4) containing 2.5% glutaraldehyde overnight at 4 °C, and the fixed sperm specimens were dropped onto a tiny microslide. Then a stepwise ethanol dehydration (10, 30, 50, 70, 80, 90, 100, 100%, 10 min each step) and a stepwise tert-butyl alcohol dehydration (tert-butyl alcohol: ethanol = 1:3, tert-butyl alcohol: ethanol = 1:1, tert-butyl alcohol: ethanol = 3:1, 100% tert-butyl alcohol, 10 min each step) were performed orderly. After electric conduction treatment, a S-4800 scanning electron microscope (SEM) (Hitachi High-Tech) was used to examine the sperm. Head width and tail length of the sperm were measured by SEM and ImageJ software [50]. In this study, 31 sperm from 3 males of GSD and 59 sperm from 3 males of TSD were measured for sperm head width detection, and 39 sperm from 3 males of GSD and 37 sperm from 3 males of TSD were measured for sperm tail length detection. The unpaired T-test was used to estimate the significant difference between sperm from males of GSD and TSD via SPSS software v19.0.0.

For transmission electron microscopy (TEM), the fixed sperm samples were washed in PBS and incubated with PBS containing 1% OsO4. Then the samples were treated with 50, 70, 80, 90 and 95% ethanol orderly and processed with a mixed solution of acetone and epoxy resin (1:1 for 1 h, and then 1:3 for 3 h). After infiltration with epoxy resin and ultrathin-section treatment, the samples were stained with uranyl acetate and lead citrate. The specimens were observed with a HT7700 transmission electron microscope (Hitachi High-Tech).

### Analysis of sperm vitality and hatchability

The semen was diluted 500 times by Hank’s solution, then 10 μl diluted semen was dropped onto on a glass slide. The prepared samples were observed and tested under a computer assisted sperm analyzer (CASA) system as soon as the sperm was activated by dropping 1 μl water. The parameters of sperm motility and kinematics were assessed by Animal Motility Software Manual Version 1.4. And 61,163 sperms from 3 males of GSD and 83,516 sperms from 3 males of TSD were detected in total.

The ovulated eggs of maternal gibel carp were inseminated with sperm from males of GSD and TSD, and the embryos were incubated in culture dishes at 23 °C (±1 °C) during the periods of embryogenesis and larval hatching. The hatchability was calculated as described [51], that hatchability = (the number of hatched larvae / the number of all fertilized eggs) × 100%. The unpaired T-test was used to estimate the significant difference of sperm vitality and hatchability between males of GSD and TSD via SPSS software v19.0.0.

### DAPI staining in fertilized eggs

The ovulated eggs of the females from strain A^+^ in gibel carp were inseminated by sperms from males of GSD in strain A^+^, from males of TSD in strain A^+^, and from another species common carp. The fertilized eggs were digested by 0.25% trypsin to remove their shells and then incubated at 23 °C for cytological observations. The fertilized eggs of different developmental stage were fixed with 4% paraformaldehyde in PBS at 4 °C overnight. After washing with PBS three times, the nuclei were stained by DAPI, and the images were acquired under confocal microscopy (NOL-LSM 710 Carl Zeiss) as described [19].

### iTRAQ-based quantitative proteomics

Total proteins of 6 semen samples from 3 males of GSD and 3 males of TSD in strain A^+^ were extracted respectively as described before [52, 53], and the protein samples were quantified by Bradford Assay and SDS-PAGE analysis. The extracted proteins were digested using Trypsin Gold (Promega) after being diluted by 100 mM triethylamine borane. Then peptides were labeled using iTRAQ Reagent8-plex Kit (AB SCIEX) according to the manufacturer’s protocol [54]. The labeled peptides with different reagents were combined and desalted with a Strata X C18 column (Phenomenex) and vacuum-dried. Subsequently, the peptides were separated on a LC-20AB HPLC Pump system (Shimadzu), and the LC-MS/MS analysis was carried out as described [55].

The raw MS/MS data was converted into MGF format by ProteoWizard tool msConvert, the exported MGF files were searched in National Center for Biotechnology Information (NCBI) (https://www.ncbi.nlm.nih.gov/) and the Universal Protein Resource (UniProt) (http://www.uniprot.org/) using MASCOT version 2.3.02 (Matrix Science) and at least one unique peptide was necessary for the identified protein. Gene Ontology (GO) database (http://www.geneontology.org/) [56], Clusters of Orthologous Groups of proteins (COG) database (http://www.ncbi.nlm.nih.gov/COG/) [57] and Kyoto Encyclopedia of Genes and Genomes (KEGG) database (http://www.genome.jp/kegg/pathway.html) [58] were used for analysis.

### Identification of differentially expressed proteins

Automated software IQuant were used for protein quantification as previously reported [59]. The peptide-spectral match (PSM) was pre-filtered at a PSM-level false discovery rate (FDR) of 1% for assessing the confidence of peptides. In order to control the rate of false-positive at protein level, an protein FDR at 1% was estimated after protein inference (protein-level FDR ≤ 0.01) [60]. Proteins with 1.2 fold change and *P*-value less than 0.05 were determined as differentially expressed proteins (DEPs).

### *Reactome* database overrepresentation and KEGG pathway enrichment

Overrepresentation analyses were performed in the *Reactome* database (https://reactome.org/) using up-regulated DEPs and down-regulated DEPs. The *P*-value indicated the statistical significance of each hit pathway, the false discovery rate (FDR) was calculated for estimating the false positives via Benjamini-Hochberg approach in *Reactome* [61], FDR < 0.25 showed the confidence of “possible” or “hypothesis”, FDR < 0.05 denoted statistical significance [26]. All DEPs were used to perform KEGG pathway enrichment analysis using cluster profiler in R via Fisher’s exact test, *P* < 0.05 were considered as statistical significance.

### BrdU incorporation and immunofluorescence detection

BrdU (5-bromo-2-deoxy-uridine) dissolved in PBS with the concentration of 0.01 mg/ml was microinjected into eggs (1 nl each) within 10 min after fertilization. Then, the BrdU incorporation embryos were fixed in 4% formaldehyde, 0.25% glutaraldehyde, and 0.1% Triton X-100 in PBS at 4 °C overnight. After treating with 2 N HCl for 30 min, neutralization by 0.1 M sodium borate (pH = 8.5) and washing in PBST (PBS containing 0.1% Triton X-100), the embryos were subjected to immunofluorescence detection according to standard protocols. Mouse α-BrdU antibody was used as primary antibody, and Rhodamine conjugated goat anti-mouse IgG was used as secondary antibody. Images were acquired under confocal microscopy (NOL-LSM 710 Carl Zeiss) as described [19].

### Analysis of protein interaction network

DEPs assigned to the process of “Replication and repair”, “Translation” and “Transcription” were used to perform protein interaction network analysis via String (http://string-db.org/). Amino acid sequences of DEPs were uploaded to the String server, and database of *Danio rerio* was selected. The threshold of minimum required interaction score was set to the highest confidence (score = 0.900), and the results were visualized by using software Cytoscape3.5.1 [62].

## Additional files

Additional file 1: **Table S1.** Detail data of male incidence, related to Fig. 3. (DOC 34 kb)

Additional file 2: **Table S2.** All proteins identified by iTRAQ approach and their annotation information in 6 databases. (XLS 4444 kb)

Additional file 3: **Table S3.** Up-regulated DEPs (males of TSD vs males of GSD). (XLS 235 kb)

Additional file 4: **Table S4.** Down-regulated DEPs (males of TSD vs males of GSD). (XLS 243 kb)

Additional file 5: **Table S5.** Detailed data of the top 20 enriched KEGG pathways, related to Fig. 6b (XLS 40 kb)

Additional file 6: **Table S6.** DEPs assigned to the process of “Replication and repair”, related to Fig. 6c. (XLS 28 kb)

Additional file 7: **Table S7.** DEPs assigned to the process of “Translation”, related to Fig. 6d. (XLS 29 kb)

Additional file 8: **Table S8.** DEPs assigned to the process of “Transcription”, related to Fig. 6e (XLS 27 kb)

Additional file 9: **Table S9.** Subcellular location of down-regulated DEPs assigned to the process of “Replication and repair”, “Translation” and “Transcription”. (XLS 35 kb)

## Abbreviations

BrdU: 5-bromo-2-deoxy-uridine
CASA: computer assisted sperm analyzer
COG: Clusters of Orthologous Groups of proteins
DEPs: differentially expressed proteins
DO: dissolved oxygen
ESD: environmental sex determination
F: flagellum
FDR: false discovery rate
GO: Gene Ontology
GSD: genotypic sex determination
HE: haematoxylin–eosin
KEGG: Kyoto Encyclopedia of Genes and Genomes
M: mid-piece
MSM: male-specific marker
N: nucleus
NCBI: National Center for Biotechnology Information
NR: non-redundant protein
PBST: PBS containing 0.1% Triton X-100
PSM: peptide-spectral match
SEM: scanning electron microscope
TEM: transmission electron microscopy
TrEMBL: Translated EMBL Nucleotide Sequence Data Library
TSD: temperature-dependent sex determination
Uniprot: Universal Protein Resource
VAP: average path velocity
VCL: curvilinear velocity
VSL: straight-line velocity
