# Supplementary material for: Distinct sperm nucleus behaviors between genotypic and temperature-dependent sex determination males are associated with replication and expression-related pathways in a gynogenetic fish
Source: BMC Genomics. 2018 Jun 5;19:437. doi: 10.1186/s12864-018-4823-6 (PMC5987661; doi:10.1186/s12864-018-4823-6)
Supplement: Supplementary file 1 — Table S1. Detail data of male incidence, related to Fig. 3. (DOC 34 kb) [file 12864_2018_4823_MOESM1_ESM.doc]

**Table S1. Detail data of male incidence, related to Fig. 3**

| Propagation | A^+^♀ × A^+^♂(+) | | | A^+^♀ × A^+^♂(-) | | | A^+^♀ × Common carp ♂ | | |
| --- | --- | --- | --- | --- | --- | --- | --- | --- | --- |
|  | Female | Male | Male | Female | Male | Male | Female | Male | Male |
|  | number | number | proportion (%) | number | number | proportion (%) | number | number | proportion (%) |
| Replicate 1 | 11 | 5 | 31.30% | 7 | 0 | 0 | 45 | 0 | 0 |
| Replicate 2 | 13 | 3 | 18.80% | 33 | 0 | 0 | 38 | 0 | 0 |
| Replicate 3 | 35 | 42 | 54.50% | 54 | 0 | 0 | 80 | 0 | 0 |
| Average |  |  | 34.9%±18.1% |  |  | 0 |  |  | 0 |
